# Biological significance of long-term bisphenol A release in the saliva of patients wearing orthodontic appliances: A systematic review and meta-analysis

**DOI:** 10.4317/jced.61735

**Published:** 2024-07-01

**Authors:** Andrea Vítores-Calero, Natalia Zamora-Martínez, Carlos Bellot-Arcís, Beatriz Tarazona-Álvarez, Jose-María Montiel-Company, Verónica García-Sanz, Vanessa Paredes-Gallardo

**Affiliations:** 1 Research Fellow, Department of Orthodontics, Faculty of Medicine and Dentistry, University of Valencia, Valencia, Spain; 2 PhD Assistant Professor, Department of Orthodontics, Faculty of Medicine and Dentistry, University of Valencia, Valencia, Spain; 3 Full Professor, Department of Orthodontics, Faculty of Medicine and Dentistry, University of Valencia, Valencia, Spain; 4 Full Professor, Department of Preventive Dentistry, Faculty of Medicine and Dentistry, University of Valencia, Valencia, Spain

## Abstract

**Background:**

Orthodontic appliances contain Bisphenol A and are controversial due to its potential risks for human health. Thus, the aim of the present research was to identify the presence of Bisphenol A in the saliva of patients wearing orthodontic appliances.

**Material and Methods:**

A systematic search of the literature was conducted in four electronic databases (PubMed, Embase, Scopus, and Web of Science) and a manual search of grey literature. Research was done up to March 2023, without language restrictions. Based on inclusion/exclusion criteria, data were extracted by two independent reviewers.

**Results:**

A total of 2293 potentially eligible articles were identified, of which 8 were finally included. The studies included a total of 238 patients and showed a moderate quality in the PEDro scale. All the devices studied released Bisphenol A into the saliva, with the polycarbonate brackets being the ones that released it for a longer time. The most significant increase occurred in the first 30 minutes after bonding with composites, reaching 697 µg/g. with polycarbonate brackets.

**Conclusions:**

Although a statistically significant increase of Bisphenol A levels in the saliva of orthodontic patients were found, this increase does not exceed the maximum allowable daily intake. Thus, the use of these materials can be considered safe for human health.

** Key words:**BPA, Bisphenol-A, cytotoxicity, Orthodontic materials, Composite resins.

## Introduction

In orthodontics, bracket placement through adhesive compounds is a daily chore that requires efficient and trustworthy firm adhesion. The material sciences of resin-based composites have suffered a strong and continual advent of improvement with multiple generations of adhesive systems introduced in the mark*et al*ong the past years (1,2).

The adhesive composites in orthodontics have similar chemical composition than those of dental restorative composite materials, consisting of inorganic fillers, a solvent base and an organic matrix that most often includes glycidyl methacrylate (Bis-GMA), which breaks down into a biochemical monomer called bisphenol A (BPA) (3). Temperature and pH changes, mechanical wear, and bacterial or salivary enzymatic action accelerate this process. Apart from resin composites, this monomer is also used in the production of a wide variety of orthodontic materials such as aesthetic brackets, elastomeric ligatures, thermoplastic aligners, chains and acrylic retainers (4,5).

Despite the successful use of these materials, there is still some uncertainty about the safety and biocompatibility of some potentially harmful monomers and BPA is the most controversial one. Despite numerous discussions and a lack of consensus about its safety, BPA was classified by the European Chemicals Agency as a ‘substance of very high concern’ since it was identified as an endocrine disruptor with risks for human health (toxic for human reproduction) and environment, as determined in the Regulation Statement (6).

When adhesive composites or some orthodontic materials degrade in the mouth, BPA is released into the saliva. From saliva, passes into the rest of the body fluids, having been found in urine, adult and fetal blood, amniotic fluid, the placenta or even in breast milk (7). Its molecular structure mimics the structure of natural estrogens, producing disruptions in the endocrine system and having teratogenic effect, even at low doses. It has also been reported to accelerate the onset of puberty, to cause feminization in men, to have carcinogenic effects (breast and prostate) (8) or serve as a risk factor in human fertility. In addition, BPA could have an impact on the psychosocial health of children as it has been associated with higher levels of anxiety, depression and social stress. Apart from that, many cases of allergic contact dermatitis have also been reported among dental personnel (7).

Although sufficient medical research has proved the harmful effects of materials that release BPA, the doses usually released in the dental field do not exceed 50 mg/kg/day, which is the maximum allowable daily intake established by the United States Environmental Protection Agency (EPA) to consider it toxic or harmful (9).

Since orthodontic materials are expected to have a service life of several years in the mouth, it is necessary to investigate the long-term release of those potentially harmful ingredients Also, the debonding process may lead to the release of BPA in the saliva (5). Studies on the release of BPA from materials used in orthodontics have solely focused on identifying, *in vitro*, whether these materials have estrogenic potential or not (9). There are few studies and no reviews focused on studying the release of BPA *in vivo*, in patients wearing orthodontic appliances.

Therefore, the main objective of this work was to analyze the presence of BPA in the saliva of patients wearing orthodontic or retention appliances and to assess if the levels are within the limits reported by the international protection agencies.

## Material and Methods

-Study protocol and registration

This systematic review was conducted following the Preferred Reporting Items for Systematic Reviews and Meta-Analyses (PRISMA) statement (10). and was previously registered in PROSPERO (CRD42022380640).

The focused PICO research question was: Do orthodontic materials (I) release BPA in the saliva (O) of patients wearing orthodontics or retention appliances (P)?

-Eligibility criteria

The following eligibility criteria were applied: 1) Population: clinical trials, conducted on patients wearing orthodontic appliances. 2) Interventions: presence of orthodontic or retention appliances. 4) Outcome measure: assessment of the levels of BPA in saliva in relation to the type of material and time of exposure.

The exclusion criteria were *in vitro* studies, patients who did not wear orthodontic or retention appliances and studies about resin o sealant restorations, case reports, review and systematic review articles, retrospective studies, editorials, opinions, surveys, guidelines, conferences, commentary articles and *in vivo* animal studies.

-Information sources and search strategy

An electronic search was conducted through Pubmed, Scopus, Embase and Web of Science. In addition, a search of the grey literature was performed through Opengrey as well as a hand search of the bibliography. The search was conducted up to March 2023, with no constraints in terms of publication year or language. The search strategy is shown in  Table 1.

Two researchers (RG-B and AV-C), working independently, systematically assessed the titles and abstracts of all identified articles. If disagreement occurred, a third author (BT) was consulted. If the abstract did not contain enough information to include or exclude a particular article, the authors read the full article before making a final decision. Once potential studies were identified for inclusion, both authors retrieved and reviewed the full texts.

-Data extraction

Data extraction was performed independently and in duplicate by two authors. The following data was extracted: authors, type of study, publication year, orthodontic material, sample size, sampling times, units of measurement, sample storage temperature, technique used and results.

-Risk of bias in individual studies

The quality of clinical trials has been assessed using the Physiotherapy Evidence Database (PEDro) scale (11,12). Any disagreement between the two initial investigators was resolved by consensus and, when in doubt, a third investigator was consulted ( Table 2).

-Quantitative synthesis (meta-analysis)

The studies were combined with a random effects model using the maximum likelihood method. The effect size used was the standardized mean difference and was interpreted using the Cohen scale (d= 0.2-0.5 small effect size; d=0.5-0.8 medium; and d>0.8 significant). The forest plot shows the analysis by subgroups according to the time point. Heterogeneity was assessed with the Q test, *p* value<0.1 and by I2 (25-50% mild;50-75% moderate;>75% important).

The presence of publication bias was analyzed using the Trim and Fill study imputation method to obtain symmetry in the funnel plot and with the Egger regression intercept at *p*<0.1.

## Results

-Selection of studies

**Figure 1:**
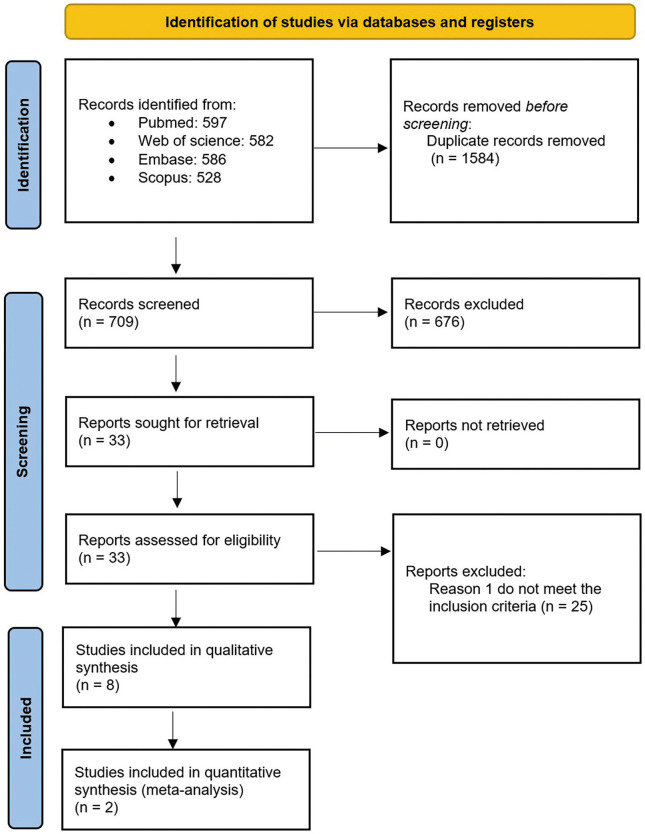
PRISMA 2020 flow diagram for new systematic reviews.

The electronic search identified 2293 preliminary references of which 597 were from Pubmed, 586 from Embase, 528 from Scopus and 582 from Web of Science. Duplicate articles were manually discarded resulting in 709 entries. From these, 676 were excluded by reading the title and abstract. After reading the resulting 33 articles, 25 were discarded because they did not meet the inclusion criteria. The reasons for rejection of the excluded articles were recorded (Supplement 1) (http://www.medicinaoral.com/medoralfree01/aop/jced_61735_s01.pdf). Finally, 8 were included for qualitative synthesis and 2 for the meta-analysis. The PRISMA 2020 flowchart (Fig. 1) provides an overview of the study selection process.

-Study characteristics

The eight studies included in this systematic review were clinical trials ( Table 3). The largest sample size was in the Görükmez *et al*. (3) study with 48 patients; the lowest occurred in the clinical trial by Watanabe *et al*. (13) analyzing 12 polycarbonate brackets from 3 patients.

Four articles (3,9,14,15), studied the release of BPA from composites used for bonding metal brackets. Watanabe *et al*. (13), analyzed aesthetic polycarbonate brackets and Raghavan *et al*. (8) and Kang *et al*. (16) focused on fixed and removable retention. Also, the study by Seifi *et al*. (5) investigated the residual adhesive removal method that released less BPA in the saliva.

Most of the studies included a follow-up period of 1 month, except for Watanabe *et al*. (13) whose follow-up was 40 months, Kloukus *et al*. (15) whose follow-up ended after the patient’s first rinse and Seifi *et al*. (5) that ended after removing the remaining adhesive. All studies analyzed BPA in saliva and the articles by Moreira *et al*. (9) and Kang *et al*. (16) also analyzed BPA in urine. The BPA measurement technique differed according to the studies, three used liquid chromatography (8,14,16), three used gas chromatography (5,9,15) and Watanabe *et al*. (13) used the scanning electron microscope.

Each study used different units of measurement and storage temperature ranged between -80º and 37º. The studies of Görükmez *et al*. (3) and Raghavan *et al*. (8), stored the samples at -20º and measured the BPA in ppb and ppm respectively. Moreira *et al*. (9), Kloukus *et al*. (15) and Kang *et al*. (16) measured the BPA in nanograms, split grams, liters or milliliters respectively. Finally, the articles of Manoj *et al*. (14) and Seifi *et al*. (5) used μg/ml and Watanabe *et al*. (13) used μg/g.

-Synthesis of the results

The two studies comparing self-curing and light-curing composites are those by Görükmez *et al*. (3) and Manoj *et al*. (14). In the article by Manoj *et al*. (14) the baseline BPA levels were of 0 μg/ml, founding a higher release from self-curing composites, with a maximum leach out of 36 μg/ml whereas the light-cured group released 19 μg/ml. Görükmez *et al*. (3) found baseline BPA levels of 0.603 ppb, with a higher release by light-curing composites (29.715 ppb) than self-curing composites (13.693 ppb). Furthermore, Görükmez *et al*. (3) found no difference between rinsing and not rinsing with water after applying the bonding.

Kang *et al*. (16), found that the group treated by the nanohybrid composite did not have detectable baseline BPA levels and in the other group treated by hybrid composite the basal levels were 0.8389 ng/ml, coinciding with the articles by Moreira *et al*. (9) and Kloukus *et al*. (15), that studied photopolymerizable composite and also started from detectable levels of BPA in saliva, 0.56 ng/g- 155 ng/l respectively.

In addition, Kang *et al*. (16) found that the hybrid composite released more BPA (7.2676 ng/ml vs. 2.3211 ng/ml) with a statistically significant difference between the groups. The study by Kloukus *et al*. (15), found statistically significant differences in the increase in BPA after bonding and in the decrease in BPA after the water rinse, but not with the water/ethanol rinse. The studies by Moreira *et al*. (9) and Kang *et al*. (16) agreed that the greatest release of BPA occurs 30 minutes after bonding, but Kang *et al*. (16) did not find detecTable levels per month and Moreira *et al*. (9), yes. Raghavanan *et al*. (8), found that essix retainers released more BPA than self-cured Hawley retainers followed by heat-cured Hawley retainers. On the other hand, the highest levels of BPA released by the essix retainer were detected after a week and with the Hawley this happened after their placement. Watanabe *et al*. (13) found a progressive increase of BPA levels in saliva after bonding polycarbonate brackets, even 40 months later. Furthermore, the release of this monomer was higher *in vivo* than *in vitro*.

-Risk of Bias

The articles have obtained moderate quality on the PEDro scale ( Table 2). This scale is made up of 11 items, with 11 being the highest score. However, item 9 has not been assessed, due to the absence of a control group because the studies compare the initial values of BPA and the values after inserting the orthodontic device in the same patient. Thus, the maximum possible score is 10. Two studies obtained 6/10 (14,15), two studies 5/10 (3,8) and three 4/10 (9,13,16). The study by Seifi *et al*. (5) obtained the highest score because it is the only one that meets item number 7 that corresponds to the masking of the evaluators.

-Quantitative synthesis (meta-analysis)

Two studies (9,14) with four time points have been combined using a random effects model. The estimated effect size was the standardized mean difference (d) between the intervention group and the control group at each of the four time points using a subgroup meta-analysis.

**Figure 2:**
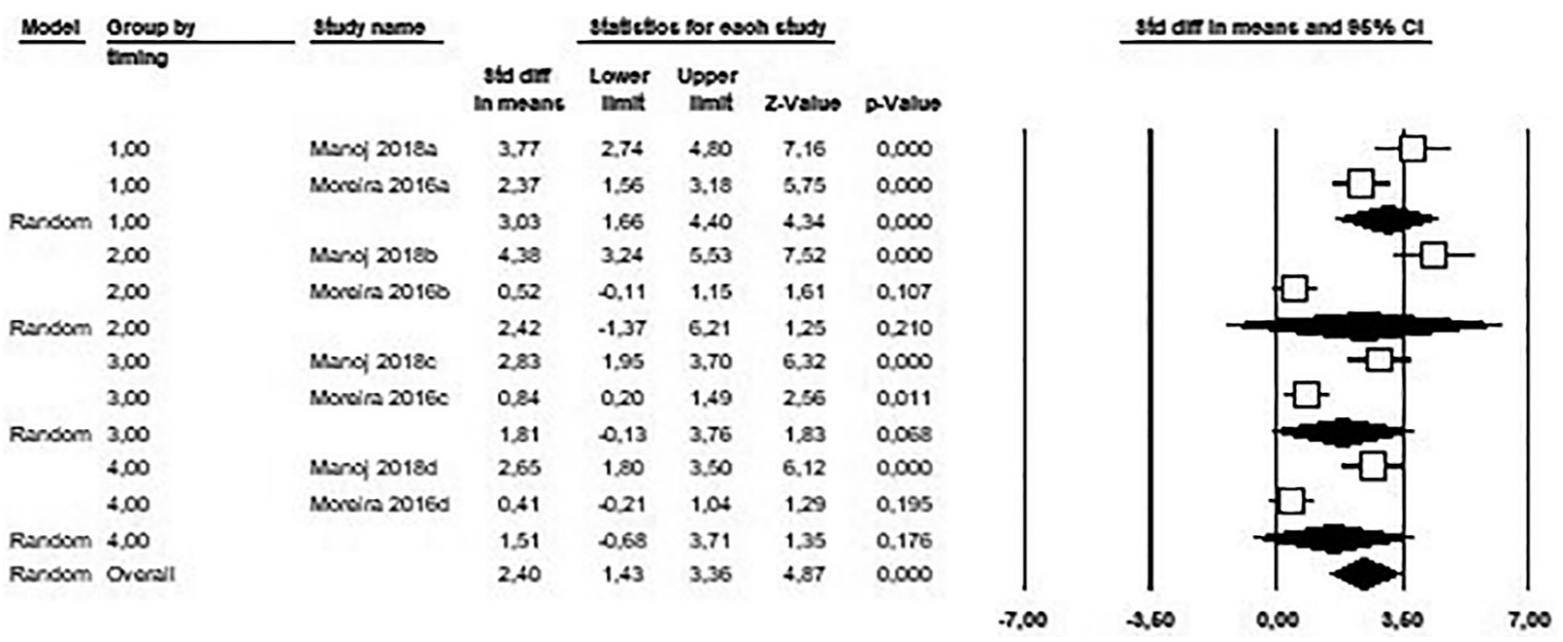
Forest Plot of the included studies.

Figure 2 shows how a release of BPA has been estimated with an effect size at time point 1 (30 minutes after the brackets were bonded) of 3.03 (CI 1.66-4.40). In this way, and according to the Cohen scale, we found an important effect size, being statistically significant (*p* value < 0.001). The effect size decreases over time, ceasing to be statistically significant. Thus, at time point 2 it is 2.42 (IC -1.37-6.21), at time point 3 it is 1.81 (IC -0.13-3.76) and finally at time point 4 it is 1.51 (IC -0.68 -3.71).

The meta-analysis has presented significant heterogeneity with a Q test *p* value <0.001 and I2 = 91.9%.

-Publication Bias

**Figure 3:**
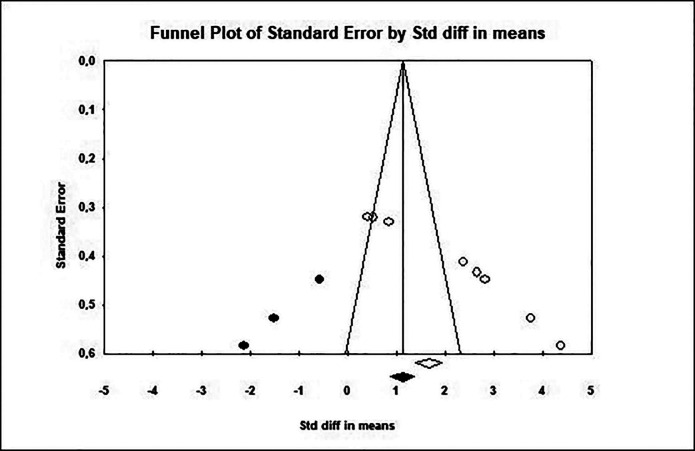
Funnel Plot Trim and Fill.

Through the trim and fill analysis (Fig. 3), we can observe the imputation of 3 studies to the left of the funnel plot to form a symmetrical image, resulting in a new estimate that differs significantly from the initial estimate. In addition, with Egger’s regression analysis, we obtained an intercept value of 15.9 (CI 13.4-18.4), statistically significant with a *p* value <0.001. For all these reasons, we can affirm that the meta-analysis is exposed to a possible publication bias.

## Discussion

Even though BPA is present in most of the composites used for bonding brackets and retainers, the literature on the release of BPA in saliva is still limited. Although this monomer has been found to be an estrogenic disruptor that can have serious repercussions at high doses causing potentially health problems, is still unclear whether its presence in dental composites can have a harmful effect.

There is published literature related to the release of BPA from orthodontics materials, such as the articles of Iliadi *et al*. (4) and Halimi *et al*. (7), but these reviews included both *in vivo* and *in vitro* studies. In this systematic review it was decided to include only *in vivo* studies, since the study by Watanabe *et al*. (13), found that BPA was released 10 times more *in vivo* than *in vitro*. So, the inclusion of *in vitro* studies could lead to underestimating the levels of BPA.

The systematic review of Iliadi *et al*. (4), focused on analyzing the cytotoxic effects and the release of BPA only from thermoplastic aligners and retainers, unlike the present review that analyzed all the materials used in orthodontics. The only article included in both reviews was the one of Raghavan *et al*. (8). On the contrary, the systematic review of Halimi *et al*. (7) included 21 articles, of which 10 were *in vitro* studies and 11 studied the release of BPA from orthodontic materials. Only the studies of Kloukus *et al*. (15) and Kang *et al*. (16), were *in vivo* studies and both have also been included in the present review.

The studies have a medium/low quality and none of them meets items 3,5,6, which correspond to blinding, since both the professionals and the participants knew the group to which each patient belonged. Only the study by Seifi *et al*. (5), who achieved the highest score, meets item 7, which corresponds to the masking of the evaluators.

Secondly, the study with the longest follow-up was the one of Watanabe *et al*. (13). These authors estimated that the release of BPA from polycarbonate brackets over 34 months was 374 μg/g. This value corresponded to 8.2 μg per bracket (bracket weight, 22 mg). If 10 brackets were applied to a 40 kg body weight patient for 34 months, then the total BPA intake would be 82 μg or a daily intake of 2.0 ng/kg/day. This value is 100 times lower than the maximum non-estrogenic effect level of BPA of 200 ng/kg/day, whose value was calculated in mice. That is, even if 10 times more BPA were released *in vivo* than *in vitro*, that amount would still have little or no estrogenic effect.

BPA was found in restorative materials such as composites and sealants, so a patient with many composite fillings or fissure sealants would be expected to have higher levels of BPA in saliva, especially if these fillings are recent. Some studies (3,8,9,15,16), started from detecTable levels of BPA in saliva. Despite this, only three studies (5,14,15) included patients without composite restorations but even between authors there were discrepancies. Manoj *et al*. (14) found no BPA in the saliva before braces were placed, Kloukus *et al*. (15) found initial levels of 155 ng/l for the ethanol + water rinse group and 130 ng/l for the tap water rinse group and for Seifi *et al*. (5) the basal values were not reflected. This difference may be due to the selection of the sample, the different storage temperatures and the different techniques used. In Manoj *et al*. (14) and Seifi *et al*. (5) studies, patients who smoked or chewed tobacco, with kidney or liver diseases and with acrylic work or resin restorations were excluded, whereas in the Kloukus *et al*. (15) study, the exclusion criteria included only the presence of resin or sealant restorations. Aspects such as the consumption of hot drinks in plastic cups and environmental exposure to BPA should also be considered, as Manoj *et al*. (14) reported.

Kloukus *et al*. (15) showed that the oxygen inhibited layer composed of unreacted monomer species could dissolve in saliva or rinsing media and released intraorally. According to these authors, the removal of the oxygen inhibited layer immediately after bonding should be considered mandatory. Pumice prophylaxis of air-exposed cured surfaces has been shown to reduce BPA release in sealants and resin composites used as orthodontic retainers.

According to the study by Görükmez *et al*. (3), the repositioning of incorrectly placed brackets can make patients more exposed to additional residual monomers. Therefore, indirect cementation with a self-curing composite would be preferable, because, according to their study, they release less BPA than light-curing composites. In contrast Manoj *et al*. (14) did not find any difference. Such difference could be explained by the different storage temperatures of the samples used, since, as mentioned above, BPA is temperature sensitive. Also, the study by Seifi *et al*. (5) demonstrated that removal of residual adhesive by the tungsten carbide bur instead of the ultrasonic scaler can released less BPA in the saliva and is a faster method (1.008 µg./mL vs. 2.83 µg./mL). This must be considered to minimize the intake of BPA by our patients.

The study by Görükmez *et al*. (3), concluded that the use of a chromatographic technique in tandem with mass spectrometry provided more precise and sensitive results. While gas or liquid chromatography devices alone can measure down to 1 ppm (one millionth), chromatography combined with mass spectrometry can measure down to 1 ppd (one billionth). Despite this, the studies by Raghavan *et al*. (8) and Watanabe *et al*. (13) did not use this combination of techniques. Manoj *et al*. (14), used high-performance liquid chromatography to avoid bias, since, although it is a relatively slower technique, high-performance liquid chromatography can be performed at room temperature, which allowed the analysis of heat-sensitive compounds like BPA, since it can be affected by changes in oral temperature over time and gas chromatography is performed at elevated temperatures. This leads us to understand that the studies that used the liquid chromatography in tandem with mass spectrometry would obtain more reliable results than those that used gas chromatography.

Regarding the limitations of this systematic review, we found few *in vivo* studies on the subject and diversity in methodology. The comparisons between studies were difficult to perform since each study used a different unit, technique, and storage temperature.

In sum, this study provides relevant information regarding the release of BPA of different materials: is maximum after cementing metal brackets or retainers and decreases with time; thermoplastic retainers release higher levels of BPA and for a longer time than the hawley type retainers. Removal of remaining adhesive with a tungsten carbide bur or rinsing with water after bonding can help to reduce BPA to baseline levels, although in the case of the aesthetic polycarbonate brackets the release of BPA remains active until they are removed. The amount of BPA released by these dental materials has not been clinically related to harmful effects on human health.

## Figures and Tables

**Table 1:** Search Strategy.

Pubmed	("bisphenol a" OR "BPA") AND ("orthodontics" OR "orthodontic")
Scopus	TITLE-ABS-KEY (("bisphenol a" OR "BPA") AND ("orthodontics" OR "orthodontic"))
Embase	('bisphenol a'/exp OR'bisphenol a'OR'bpa') AND ('orthodontics'/exp OR'orthodontics'OR'orthodontic'/exp OR'orthodontic')
Web of Science	TOPIC ("bisphenol a" OR "BPA") AND ("orthodontics" OR "orthodontic")

**Table 2:** Quality of clinical trials on the PEDro scale.

AUTHOR	1	2	3	4	5	6	7	8	9	10	11	Total(0-10)
Manoj et al. (14)	*	*		*				*	-	*	*	6
Raghavan et al. (8)	*	*						*	-	*	*	5
Moreira et al. (9)	*							*	-	*	*	4
Kloukos et al. (15)	*	*		*				*	-	*	*	6
Kang et al. (16)	*							*	-	*	*	4
Watanabe et al. (13)	*							*	-	*	*	4
Görükmez et al. (3)	*	*						*	-	*	*	5
Seifi et al. (5)	*	*		*			*	*	-	*	*	7

**Table 3:** Main characteristics of the studies.

Author /study type (year)	Orthodontic supplies	Sample size	Sampling Time / BPA Measurement Units	Technique for measuring BPA/storage temperature	Results
Manoj et al. (14) (2018) Clinical Trial	Composite photopol and autopol bonded metal brackets	40 2 groups	0, 30min, 1day, 1week, 1month 0= before bonding μg/ml	liquid chromatography and mass spectrometry -	BPA release: autopol > photopol Maximum release at 30 min, after which it drops, with release still remaining after a month
Raghavan et al. (8) (2017) Clinical Trial	Essix Hawley (heat) Hawley (chemical)	45 3 groups	0, 1h, 1week, 1 mes ppm	liquid chromatography -80º	BPA release: Essix > Hawley (chemical cure) > Hawley (heat cure) Max BPA: Essix 1wk, Hawley 1h
Moreira et al. (9) (2016) Clinical Trial	Composite photopol bonded metal brackets	20	0, 30min, 1day, 1week, 1month ng/g	gas chromatography and mass spectrometry 37º	Saliva SS increase of BPA in saliva at 30 min. In the rest of T it is higher than T0 but without being SS
Kloukus et al. (15) (2015) Clinical Trial	Composite photopol bonded metal brackets	20 2 groups 1 water 2 water + ethanol	T0 T1 bonding T2 first rinse ng/l	gas chromatography and mass spectrometry 4º	BPA increases at T1 and decreases at T2 BPA release after rinsing: Water > water + ethanol
Kang et al. (16) (2011) Clinical Trial	Lingual retainer	22	0, 30min, 1day, 1week, 1month ng/ml	liquid chromatography and mass spectrometry -80º	Saliva SS increase of BPA in saliva immediately after retainer cementation. Later, values similar to the initial one are obtained.
Watanabe et al. (13) (2004) Clinical Trial	Polycarbonate brackets	3 patients 12 brackets	18, 25, 30 and 40 months μg/g	scanning electron microscope -	BPA increases over time, being maximum at 40 months
Görükmez et al. (3) (2021) Clinical Trial	Composite photopol and autopol bonded metal brackets	48 2 groups	T0 T1 bonding T2 first rinse Ppb	liquid chromatography and mass spectrometry -20º	Higher amount of BPA in light-curing adhesives. There was no SS difference in Bis-GMA concentration at T1 and T2 (p>0.05).
Seifi et al. (5) (2023) Clinical Trial	Adhesive removal method: -tungsten carbide bur - ultrasonic scaler	40 2 groups	Before debonding After removing the remaining adhesive μg/ml	Liquid chromatography and mass spectrometry 4º	Residual adhesive removal with tungsten carbide bur released less BPA in saliva and shortened the adhesive cleaning time than the ultrasonic scaler method.

Autopol= autopolymerizable; BPA= bisphenol A; Photopol= photopolymerizable; Min= minutes; SS= statistically significant; T= time; Tº= temperature

## Data Availability

The datasets used and analyzed in the current study are available from the corresponding author upon reasonable request.

## References

[1] Dallel I, Lahwar S, Jerbi MA, Tobji S, Ben Amor A, Kassab A (2019). Impact of adhesive system generation and light curing units on orthodontic bonding: In vitro study. Int Orthod.

[2] Malhotra N, Mala K (2010). Light-curing considerations for resin-based composite materials: a review. Part I. Compend Contin Educ Dent.

[3] Görukmez E, Sen Yilmaz B, Ramoglu SI (2021). Is a Single Rinse Effective on Evacuating the Residual Monomers After Orthodontic Bonding? An In Vivo Study. Bezmiâlem Sci.

[4] Iliadi A, Koletsi D, Papageorgiou SN, Eliades T (2020). Safety Considerations for Thermoplastic-Type Appliances Used as Orthodontic Aligners or Retainers. A Systematic Review and Meta-Analysis of Clinical and In-Vitro Research. Materials (Basel).

[5] Seifi S, Mirzakouchaki B, Rafighi A, Aghanejad A, Hamidi AA, Shahrbaf S (2023). Evaluation of the bisphenol released in the saliva after residual adhesive removal in orthodontic patients by using ultrasonic scaling and rotary system: A single-center randomized clinical trial. Am J Orthod Dentofacial Orthop.

[6] De Nys S, Putzeys E, Duca RC, Vervliet P, Covaci A, Boonen I (2021). Long-term elution of bisphenol A from dental composites. Dent Mater.

[7] Halimi A, Benyahia H, Bahije L, Adli H, Azeroual MF, Zaoui F (2016). A systematic study of the release of bisphenol A by orthodontic materials and its biological effects. Int Orthod.

[8] Raghavan AS, Sathyanarayana HP, Kailasam V, Padmanabhan S (2017). Comparative evaluation of salivary bisphenol A levels in patients wearing vacuum-formed and Hawley retainers: An in-vivo study. Am J Orthod Dentofacial Orthop.

[9] Moreira MR, Matos LG, de Souza ID, Brigante TA, Queiroz ME, Romano FL (2017). Bisphenol A release from orthodontic adhesives measured in vitro and in vivo with gas chromatography. Am J Orthod Dentofacial Orthop.

[10] Liberati A, Altman DG, Tetzlaff J, Mulrow C, Gøtzsche PC, Ioannidis JPA (2009). The RPISMA statement for reporting systematic reviews and meta-analyses of studies that evaluate health care interventions: explanation and elaboration. J Clin Epidemiol.

[11] Macedo LG, Elkins MR, Maher CG, Moseley AM, Herbert RD, Sherrington C (2010). There was evidence of convergent and construct validity of Physiotherapy Evidence Database quality scale for physiotherapy trials. J. Clin. Epidemiol.

[12] Maher CG, Sherrington C, Herbert RD, Moseley AM, Elkins M (2003). Reliability of the PEDro scale for rating quality of randomized controlled trials. Phys. Ther.

[13] Watanabe M (2004). Degradation and formation of bisphenol A in polycarbonate used in dentistry. J Med Dent Sci.

[14] Manoj MK, Ramakrishnan R, Babjee S, Nasim R (2018). High-performance liquid chromatography analysis of salivary bisphenol A levels from light-cured and chemically cured orthodontic adhesives. Am J Orthod Dentofacial Orthop.

[15] Kloukos D, Sifakakis I, Voutsa D, Doulis I, Eliades G, Katsaros C (2015). BPA qualtitative and quantitative assessmentassociated with orthodontic bonding in vivo. Dental Materials.

[16] Kang YG, Kim JY, Kim J, Won PJ, Nam JH (2011). Release of bisphenol A from resin composite used to bond orthodontic lingual retainers. Am J Orthod Dentofacial Orthop.

